# Editorial: Nanomaterial-based biosensors, diagnosis, and applications

**DOI:** 10.3389/fbioe.2024.1414746

**Published:** 2024-04-24

**Authors:** Kang Cui, Li Wang, Yizhong Huang

**Affiliations:** ^1^ School of Chemistry and Chemical Engineering, University of Jinan, Jinan, China; ^2^ College of Chemistry and Chemical Engineering, Jiangxi Normal University, Nanchang, China; ^3^ School of Materials Science and Engineering, Nanyang Technological University, Singapore, Singapore

**Keywords:** nanomaterials, biosensors, diagnosis, health monitoring, biotechnology

Owing to their distinctive chemical compositions and diverse biological functionalities, numerous nanomaterials derived from organic, inorganic, and hybrid compounds have garnered significant attention from the scientific community and have played a pivotal role in advancing biosensors, diagnostics, and related applications over recent decades. These varieties of nanomaterials serve as valuable platforms for immobilization, optical interrogation, and (photo)electrochemical labeling, thereby enhancing the sensitivity, stability, and selectivity of biosensing devices, which undoubtedly reshapes conventional approaches to health monitoring, food safety, and environmental research. Accordingly, in anticipation of forthcoming advancements in this research area, we have curated this Research Topic to present the latest developments and perspectives concerning nanomaterial-based biosensors, diagnosis, and applications. Encompassing two reviews and eight original research articles, this collection spans fundamental nanomaterial physics, manufacturing techniques, and biosensing applications.

As nanomaterials play a significant role in enhancing the performance of electrochemical biosensors due to their unique physical and chemical properties, such as large surface area, high catalytic activity, and superior electron transfer kinetics. Shahid et al. highlights the application of carbon-based nanomaterials, metallic nanoparticles, quantum dots, and nanowires in biosensors. In this review article, they summarized a range of fabrication strategies utilizing diverse nanocomposites which enhance the limit of detection for different miRNAs and advance the miniaturization process of electrochemical biosensors. This review article offers researchers a comprehensive perspective on the advancements in electrochemical biosensors, particularly focusing on the latest strategies for improving detection limits and biosensor miniaturization. As the synergy between nanomaterials with biomolecules is also a key part to improve the performance of biosensors, Reaño et al. review the synergistic systems of antibodies, aptamers, and nanomaterials for amplified electrochemical signaling. The review discusses how antibodies, aptamers, or target antigens can be immobilized on the electrode, and the importance of proper immobilization techniques to support the formation of bioreceptor-biomarker complex and induce signal generation.

In the original work, Toyos-Rodríguez et al. emphasize the significance of electrochemical techniques in the development of highly sensitive biosensors by optimizing the thickness of nanoporous alumina membranes which are integral to the electrochemical sensing platform for the detection of catalase, a key biomarker in wound infection. The study successfully applies this optimization to construct a label-free immunosensor that effectively detects catalase with high precision, showcasing the potential of electrochemical methods for rapid and accurate diagnostics in clinical settings. The research article by Cheng et al. focuses on the development of a photoelectrochemical (PEC) biosensor based on SiW_12_@CdS quantum dots and colloidal gold nanoparticles, providing a low background signal and good sensitivity, for the detection of HPV 16 DNA, a significant biomarker for cervical cancer. The study highlights the importance of the PEC technique in advancing cancer diagnostics and the potential for early detection, which is critical for improving treatment outcomes. In the original work by Kan et al. the importance of the fluorescent technique is highlighted by its ability to enable real-time monitoring and selective detection of SO_3_^2−^ at low concentrations within complex biological environments, allowing for the rapid, sensitive, and selective detection of sulfate ions, which is crucial for understanding cellular processes, diagnosing diseases, and guiding drug development. The research highlights the importance of fluorescent probes in advancing biomedical studies and emphasizes their potential practical application in clinical settings. In the research article, Nishan et al. present the development of a colorimetric biosensor for detecting H_2_O_2_ using zinc oxide nanoparticles deposited on Morus nigra sawdust. This colorimetric technique allows for the visual detection of H_2_O_2_ through a change in color facilitated by the presence of 3,3′,5,5′-tetramethylbenzidine. The advantages of the colorimetric technique render it a valuable tool in biotechnology, medical diagnostics, and environmental monitoring. The original research by Cheng et al. concentrates on the design and fabrication of a surface-enhanced Raman scattering (SERS) sensor based on functionalized Au/Si cap-cone arrays and Au nanocubes modified with 5-carboxyfluorescein as a probe tailored for the highly sensitive detection of Vimentin, a protein linked to gastric cancer. The study elucidates the utility of SERS as a valuable technique for the early detection of cancer and for monitoring therapeutic responses. Besides, the research article by Lv et al. focuses on the development of a highly sensitive flexible capacitive pressure sensor that utilizes a hierarchical pyramid micro-structured polydimethylsiloxane dielectric layer. The research demonstrates the potential of flexible sensors in advancing technology for electronic skin, intelligent robotics, and personalized health monitoring.

A multiplex detection biosensor, which is capable of detecting or measuring biological analytes through multiple modes or strategies simultaneously or sequentially, can enhance the versatility, sensitivity, and reliability of the biosensor. The original works from Xiao and Wang focused on the multiplex detection techniques. Xiao et al. combines the graphene oxide-assisted multiplex recombinase polymerase amplification assay, targeting two conserved insertion sequences (IS6110 and IS1081) within *mycobacterium tuberculosis* complex (MTBC), with a CRISPR–Cas12a-based trans-cleavage assay for a comprehensive diagnostic approach. The combined approach with a CRISPR–Cas12a-based multiplex detection aims to provide a straightforward, expeditious, highly sensitive, and precisely targeted diagnostic tool for MTBC. Besides, Wang et al. explores a cellulase protected fluorescent gold nanoclusters which serve as dual-functional nanoclusters, demonstrating their potential as fluorescent bioprobes for ascorbic acid detection and bacterial labeling, thereby facilitating medical diagnosis and human health maintenance.

In conclusion, from the reviews and research works included in this Research Topic, it is revealed that a multitude of nanomaterial-based methodologies utilizing electrochemistry, PEC, fluorescence, SRES, and colorimetric analysis have been devised. These techniques combined with functional nanomaterials form the basis for the fabrication of different types of nanomaterial-based biosensors and diagnosis applications. However, meeting the increasing demand for application platforms characterized by high sensitivity, cost-effectiveness, and ease of operation remains a significant challenge, particularly in the rigorous fabrication standards demanded by industry. To further promote the development of biosensors, more bio-compatible nanomaterials and assembly techniques are in great demand. We believe further research in the nanomaterial-based biosensors and diagnosis will have a significant impact on future personalized healthcare, food safety and environmental analysis.

